# Porphyrin–Schiff Base Conjugates Bearing Basic Amino Groups as Antimicrobial Phototherapeutic Agents

**DOI:** 10.3390/molecules26195877

**Published:** 2021-09-28

**Authors:** María E. Pérez, Javier E. Durantini, Eugenia Reynoso, María G. Alvarez, María E. Milanesio, Edgardo N. Durantini

**Affiliations:** 1 IDAS-CONICET, Departamento de Química, Facultad de Ciencias Exactas, Físico-Químicas y Naturales, Universidad Nacional de Río Cuarto, Ruta Nacional 36 Km 601, Río Cuarto X5804BYA, Córdoba, Argentina; meperez@exa.unrc.edu.ar (M.E.P.); ereynoso@exa.unrc.edu.ar (E.R.); malvarez@exa.unrc.edu.ar (M.G.A.); 2 IITEMA, Departamento de Química, Facultad de Ciencias Exactas, Físico-Químicas y Naturales, Universidad Nacional de Río Cuarto, Ruta Nacional 36 Km 601, Río Cuarto X5804BYA, Córdoba, Argentina; jdurantini@exa.unrc.edu.ar

**Keywords:** porphyrin, Schiff base, photodynamic inactivation, reactive oxygen species, photoinactivation, microorganism

## Abstract

New porphyrin–Schiff base conjugates bearing one (**6**) and two (**7**) basic amino groups were synthesized by condensation between tetrapyrrolic macrocycle-containing amine functions and 4-(3-(*N*,*N*-dimethylamino)propoxy)benzaldehyde. This approach allowed us to easily obtain porphyrins substituted by positive charge precursor groups in aqueous media. These compounds showed the typical Soret and four Q absorption bands with red fluorescence emission (Φ_F_ ~ 0.12) in *N*,*N*-dimethylformamide. Porphyrins **6** and **7** photosensitized the generation of O_2_(^1^Δ_g_) (Φ_Δ_ ~ 0.44) and the photo-oxidation of L-tryptophan. The decomposition of this amino acid was mainly mediated by a type II photoprocess. Moreover, the addition of KI strongly quenched the photodynamic action through a reaction with O_2_(^1^Δ_g_) to produce iodine. The photodynamic inactivation capacity induced by porphyrins **6** and **7** was evaluated in *Staphylococcus aureus*, *Escherichia coli*, and *Candida albicans*. Furthermore, the photoinactivation of these microorganisms was improved using potentiation with iodide anions. These porphyrins containing basic aliphatic amino groups can be protonated in biological systems, which provides an amphiphilic character to the tetrapyrrolic macrocycle. This effect allows one to increase the interaction with the cell wall, thus improving photocytotoxic activity against microorganisms.

## 1. Introduction

Photodynamic therapy (PDT) represents a modality for the treatment of malignant tumors [1]. This technique is currently being extended to several non-cancerous illnesses, especially those characterized by unwanted cell overgrowth such as various dermatological diseases, benign prostatic hyperplasia, and age-related muscle degenerations. In the last few decades, research and development in this field have allowed for the use of photosensitizers (PSs) in the treatment of infectious diseases. This methodology is called the photodynamic inactivation (PDI) of microorganisms. In this sense, PDI could become a tool solve these types of health problems. PDI therapy is based on three fundamental stages: the preferential accumulation of the PS in the microbial cells, the illumination of the affected area to generate reactive oxygen species (ROS), and the reaction of these intermediates with the macromolecules of the cells, thus causing lethal damage [2].

A variety of macrocycles derived from porphyrins, chlorines, bacteriochlorins, and phthalocyanines have been proposed as potential phototherapeutic agents [3,4,5]. In particular, the use of porphyrins have three main advantages: high absorption in the visible area of the spectrum, chemical stability due to their aromatic structure, and low toxicity in the dark. Porphyrin synthesis is of utmost importance because it involves a wide spectrum of scientific applications [6]. The arrangement of different substituents on the periphery of the macrocycle can be used to vary its properties. The development of porphyrin synthesis strategies has enabled the obtainment of tetrapyrrolic rings with different substitution patterns. Thus, porphyrins with AB_3_ symmetry can be conveniently synthesized from the condensation of an appropriate dipyrromethane and a binary mixture of aldehydes using acid catalysis [7,8]. However, porphyrins with AABB symmetry can be obtained from pyrrole and a binary mixture of aldehydes. The formation of porphyrins asymmetrically substituted by lipophilic and hydrophilic groups can be used to generate an intramolecular axis of polarity. This amphiphilic character makes it possible to improve the interaction of porphyrins with the cell membrane [9]. In this sense, Schiff bases represent an important class of organic substituents that have attracted attention in the pharmaceutical and medical fields [10]. A wide variety of heterocyclic rings substituted by Schiff bases have a broad spectrum and a wide variety of biological activities, including antiviral, anticancer, cytotoxic, antimicrobial, antibacterial, and anticonvulsant. Therefore, the development and synthesis of new Schiff base derivatives as potential chemotherapeutic agents is currently attracting the attention of organic and medicinal chemistry. Schiff bases can be easily synthesized from the reaction of carbonyl compounds with primary amines in solution [11]. Furthermore, PS–Schiff base conjugates have been evaluated as potential phototherapeutic agents [12,13].

**Scheme 1:**
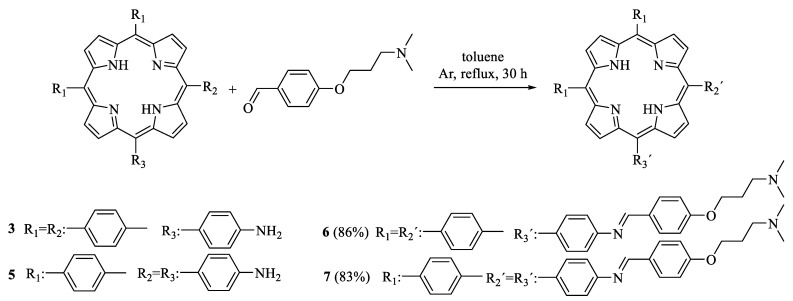
Synthesis of porphyrin–Schiff base conjugates **6** and **7**.

Here, we describe the synthesis of porphyrin–Schiff base conjugates substituted by one (**6**) or two (**7**) aliphatic amine groups (Scheme 1) as potential PSs for the PDI of microorganisms. Porphyrins substituted in the *meso* position by basic amino groups through Schiff base functions are capable of acquiring positive charges at physiological pH [14]. Thus, the presence of cationic groups from protonation can allow for better interactions with the cell wall of microbial cells [7,15,16]. In this study, the spectroscopic characteristics of porphyrins **6** and **7** were determined in solution, and their photodynamic properties were investigated in the presence of different photo-oxidizable substrates. Furthermore, the effect of KI on the decomposition of these compounds was evaluated. The application of PDI was studied in cell suspensions of the Gram-positive bacterium *Staphylococcus aureus*, the Gram-negative bacterium *Escherichia coli*, and the ySeast *Candida albicans*. *S. aureus* is the main cause of bacterial infections in humans around the world [17], strains of *E. coli* have begun to acquire substantial resistance to conventional antimicrobial treatments [18], and fungal infections produce by *C. albicans* represent one of the most prevalent fungal agents that can cause life-threatening invasive infections [19]. Therefore, developing alternative therapies to conventional antibiotics for the treatment of these infectious diseases is an interesting research topic [20]. Finally, the combined effect of PDI in the presence of KI was analyzed in order to improve the eradication of pathogenic microorganisms.

## 2. Results and Discussion

### 2.1. Synthesis of Porphyrin–Schiff Base Conjugates

The synthetic procedures to obtain porphyrin–Schiff base derivatives are shown in Scheme 1. To obtain the Schiff bases, the amino porphyrins were previously synthesized following the method reported in [21,22,23]. First, monoamide porphyrin **2** with an AB_3_ symmetry was obtained by the acid-catalyzed condensation of dipyrromethane **1** and the corresponding 4-substituted benzaldehydes in a 16% yield (Scheme S1) [21]. Then, diamide porphyrin **4** with an AABB structure was obtained from a binary mixture of benzaldehydes and pyrrole in propionic acid using a modification of the Alder–Longo method (Scheme S2) [23]. This classic procedure provides a mixture of porphyrins, from which porphyrin **4** was isolated in a low yield (1.4%). The hydrolysis of porphyrins **2** and **4** by heating in basic medium (KOH and THF/methanol) produced porphyrins with one (**3**; 76%) and two (**5**; 77%) amine functions, respectively [22,23]. After that, Schiff base derivatives **6** and **7** were synthesized by condensation between amine porphyrins **3** and **5** and 4-(3-(*N*,*N*-dimethylamino)propoxy)benzaldehyde (Scheme 1). The reaction was performed using an excess of benzaldehyde (1.0:5.4 and 1.0:7.4 porphyrin to aldehyde molar ratios for **6** and **7**, respectively) in dry toluene at reflux. The progress of the reaction was followed by TLC analysis, using dichloromethane (DCM)/methanol/triethylamine (TEA) as eluents, until the complete disappearance of the starting porphyrin. Products **6** and **7** were purified by flash column chromatography (silica gel) using a gradient elution of DCM/methanol/TEA. Basic TEA was used to prevent the decomposition of the Schiff base conjugates in the silica gel column. Subsequently, porphyrins **6** and **7** were crystallized using DCM/petroleum ether/TEA. The products were isolated as purple solids in 86% and 83% yields for **6** and **7**, respectively. A similar procedure was previously used for the synthesis of Schiff base-substituted porphyrins by reacting a porphyrin monoamine with various aldehydes containing different groups [24,25].

**Figure 1:**
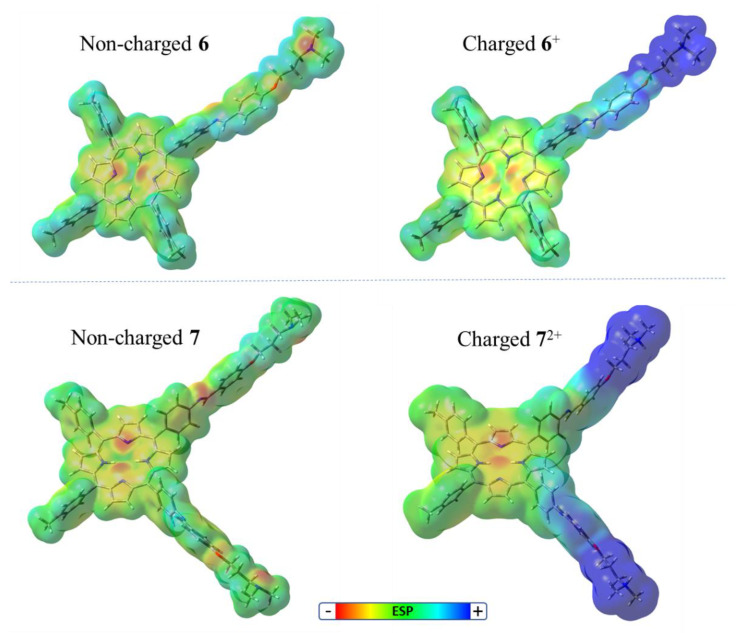
Optimized structure and the ESP surfaces of non-charged **6** and **7** and protonated analogs **6^+^** and **7^2+^** calculated for ground state by using DFT at the B3LYP/6-31G(d) level. Created in Avogadro Software founded on Gaussian 09 calculations. The positive and negative ESP regions are colored blue and red, respectively.

In the present case, both porphyrin–Schiff base conjugates **6** and **7** contain basic amino groups. These substituents can be protonated (3-(dimethylamino)propanol; pK_a_ = 10.5) [26], so they are precursors of cationic centers at physiological pH [7]. Additionally, these amino units are separated from the porphyrin macrocycle by an aliphatic spacer that affords a higher mobility. The presence of positive charges with appropriate flexibility of movements can allow for better interactions with microorganisms [7,16]. To evaluate this effect, porphyrins **6** and **7** were optimized to a stationary point on the Born–Oppenheimer potential energy surface of the molecular structures [27]. The optimized structures and ESP surfaces for the ground states of **6** and **7** were compared with that of their protonated analogs, monocationic **6^+^** and dicationic **7^2+^**, as shown in Figure 1. The phenylene unit was in an almost perpendicular arrangement with respect to the tetrapyrrolic macrocycle. From this analysis, we determined spatial regions in the molecular structure in which the molecular electrostatic potential was negative and positive. Potential surfaces showed the relative locations of partial positive and negative charges, which were colored blue and red, respectively. This study allowed us to visualize the charged regions of each molecule and the distribution in the structure of the porphyrin–Schiff base derivatives. As can be observed, non-charged **6** and **7** structures showed an almost homogeneous charge distribution, with a slight negative area on the nitrogen atoms, due to the electron-donating capacity of the aliphatic amine groups, the Schiff bases, and the center of the tetrapyrrolic macrocycle. Positive charge distributions for **6^+^** and **7^2+^** were placed around the protonated ammonium groups. Furthermore, protonated porphyrins **6^+^** and **7^2+^** showed that positive charges were highly exposed at the peripheries of the macrocycle. These results indicate that these cationic porphyrins can have strong specific electrostatic interactions with the cell walls of microbial cells [27].

The combination of the hydrophobic substituents and hydrophilic cationic groups in the PS structures produces an intramolecular polarity axis. To estimate the effect induced by the protonated substituents upon the intramolecular polarity, the dipole moment (*μ*) of porphyrins was calculated in non-charged and cationic structures (Figure S1); values of 1.98 and 2.21 D were determined for **6** and **7**, respectively. In contrast, protonated analogs **6^+^** and **7^2+^** presented values of 74.37 and 78.91 D, respectively. As a consequence, the cationic moiety attached to the periphery of tetrapyrrolic macrocycles significantly increased the value of μ with respect to the non-charged structures. The amphiphilic character of these PSs could facilitate penetration through the cell membrane, generating more accumulation in the cells and consequently increasing the PDI of microorganisms [2,4].

### 2.2. Absorption and Fluorescence Spectroscopic Properties

**Figure 2:**
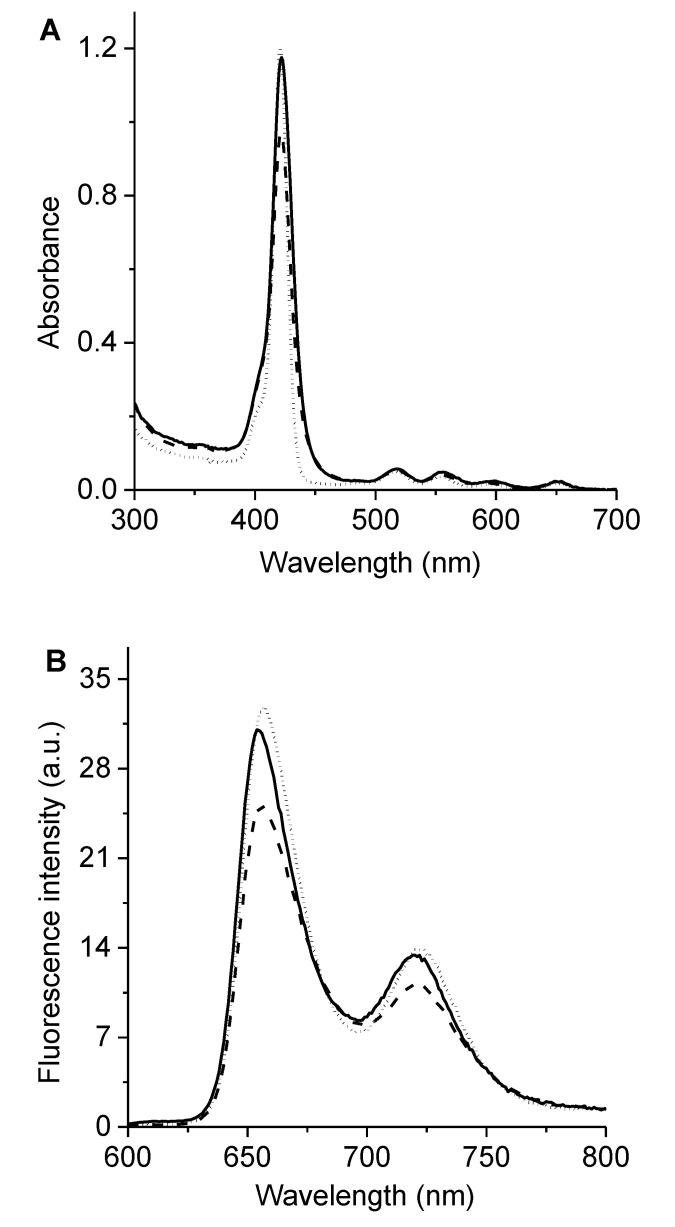

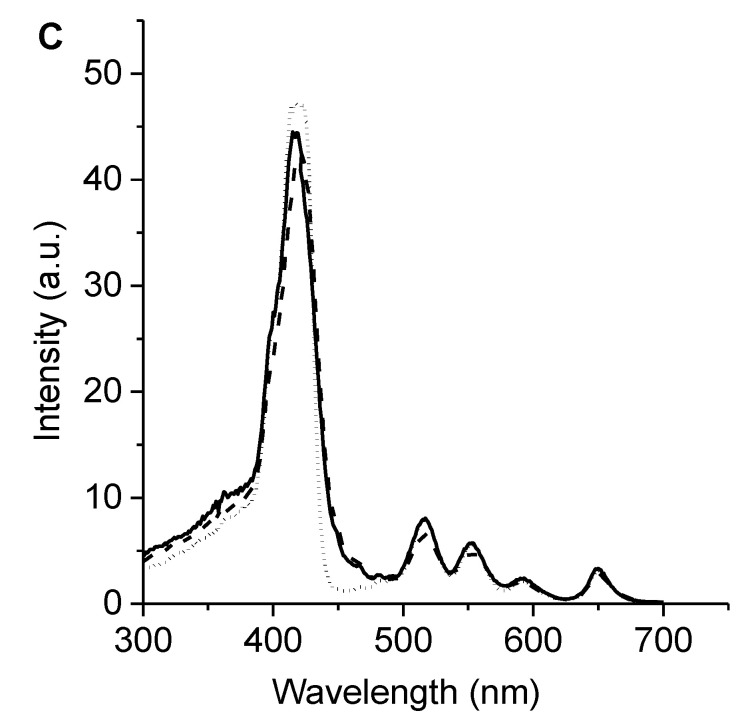
(**A**) Absorption, (**B**) fluorescence emission (*λ*_exc_ = 517 nm), and (**C**) excitation (*λ*_em_ = 720 nm) spectra of **6** (solid line), **7** (dashed line), and TMP (dotted line) in DMF.

The UV–visible absorption spectra of **6** and **7** in *N,N*-dimethylformamide (DMF) are shown in Figure 2A. These porphyrins presented a typical *ethio*-type UV–Vis spectrum composed of an intense Soret band at ~420 nm and four less intense Q bands between 500 and 670 nm, which is representative of similar porphyrin derivatives [14,28]. The Q-bands of porphyrin involves the transitions Qx(0,0), Qx(1,0), Qy(0,0), and Qy(1,0), which are related with a D_2h_ symmetry [29]. Additionally, the spectra showed sharp absorption bands, which indicated that these porphyrins were not aggregated in this medium. The observed absorption properties of porphyrins **6** and **7** were similar to that obtained for 5,10,15,20-tetrakis(4-methoxyphenyl)porphyrin (TMP), which was used as a reference (Figure 2A), indicating that the presence of Schiff base substituents does not modify the absorption spectroscopic properties of tetrapyrrolic macrocycles.

**Figure 3:**
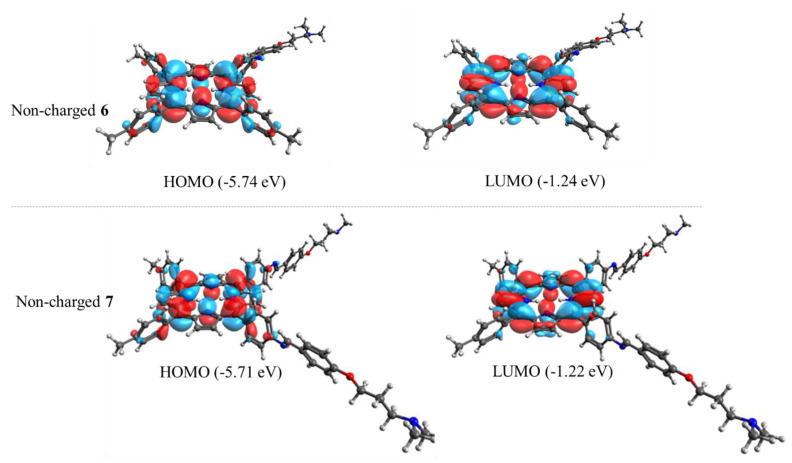
Highest occupied molecular orbital (HOMO) and lowest unoccupied molecular orbital (LUMO) of non-charged porphyrins **6** (up) and **7** (down). All calculations were performed by DFT at the CAM-B3LYP/6–31 + G(d) level using Gaussian 09.

Calculations of highest occupied molecular orbital (HOMO) and lowest unoccupied molecular orbital (LUMO) of porphyrins **6** and **7** obtained by DFT at the B3LYP/6–31 + G(d) level using Gaussian 09 are presented in Figure 3. The HOMO of both porphyrins was formed by π orbitals completely localized on the tetrapyrrolic macrocycle. These orbitals were regularly distributed over the tetrapyrrolic ring. Thus, the calculated orbitals displayed that the substituted groups produced a slight influence on the optimized structure of the tetrapyrrolic core compared to 5,10,15,20-tetrakis(phenyl)porphyrin [30]. Similar results were also obtained for protonated porphyrins **6**^+^ and **7**^2+^ (Figure S2). Therefore, this analysis shows that the major electron density distribution of HOMO and LUMO is concentrated on the porphyrin core.

The fluorescence emission of porphyrins in DMF are shown in Figure 2B. The spectra can be observed to present two bands characteristic of substituted porphyrins in the *meso* position [7,28]. These bands are centered around 655 and 720 nm and correspond to Qx(0–0) and Qx(0–1) transitions, respectively [31]. This behavior is typical of free-base porphyrins and indicates that the vibronic structure remained unchanged after excitation [29]. Furthermore, Stokes shifts of about 6 nm were calculated from the absorption and fluorescence maxima of the Qx(0–0) band. Small Stokes shifts showed that the spectroscopic energy of these porphyrins was similar to the relaxed energies of the singlet state. Thus, only minor structural changes took place between its ground and excited states, as expected due to the rigid planar structure of the tetrapyrrolic macrocycles. Moreover, the energy levels of the singlet excited stated (*E*_s_) were calculated as 1.90 eV when considering the energy of the Qx(0–0) electronic transitions from the intersection of the normalized absorption and fluorescence spectra. Additionally, the fluorescence quantum yields (Φ_F_) of these porphyrins were determined in DMF, showing of 0.13 ± 0.01 and 0.12 ± 0.01 for porphyrins **6** and **7**, respectively. The Φ_F_ values obtained for **6** and **7** agreed with those previously calculated for similar structures in this solvent [14,15]. In addition, the fluorescence excitation spectra of these porphyrins were achieved in DMF following the emission at 720 nm (Figure 2C). The excitation spectra of porphyrins **6** and **7** were similar to the absorption spectra (Figure 2A), indicating that the PSs were mainly dissolved as monomers in DMF. In previous investigations, Ya-Hong et al. studied a series of Schiff bases with different secondary substituents, and only minor changes were observed in the position of the absorption and fluorescence bands [32]. Therefore, substituents in the Schiff base groups provide weak electronic effects in π-electron systems.

### 2.3. Photo-Oxidized Decomposition of 9,10-Dimetilantraceno (DMA)

**Figure 4:**
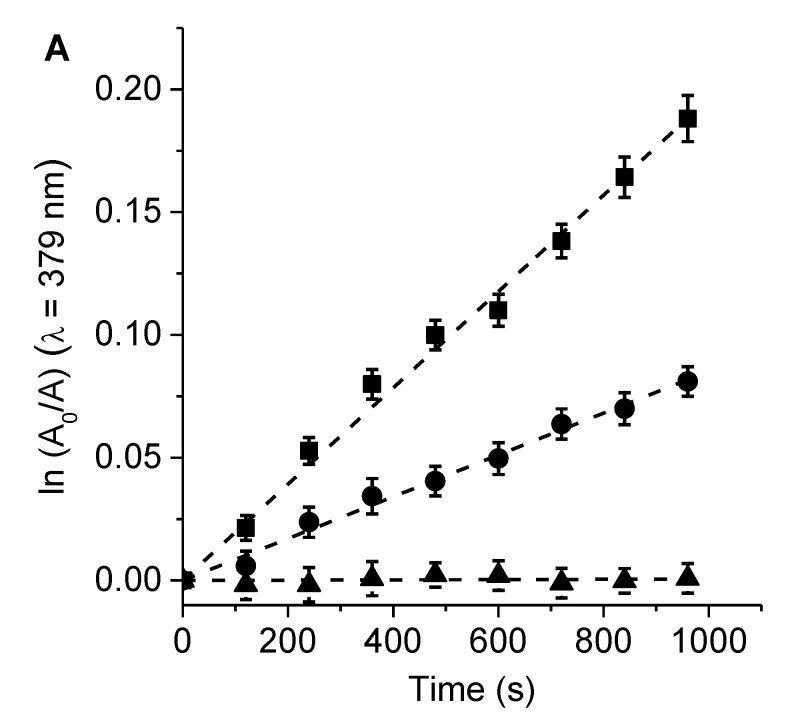

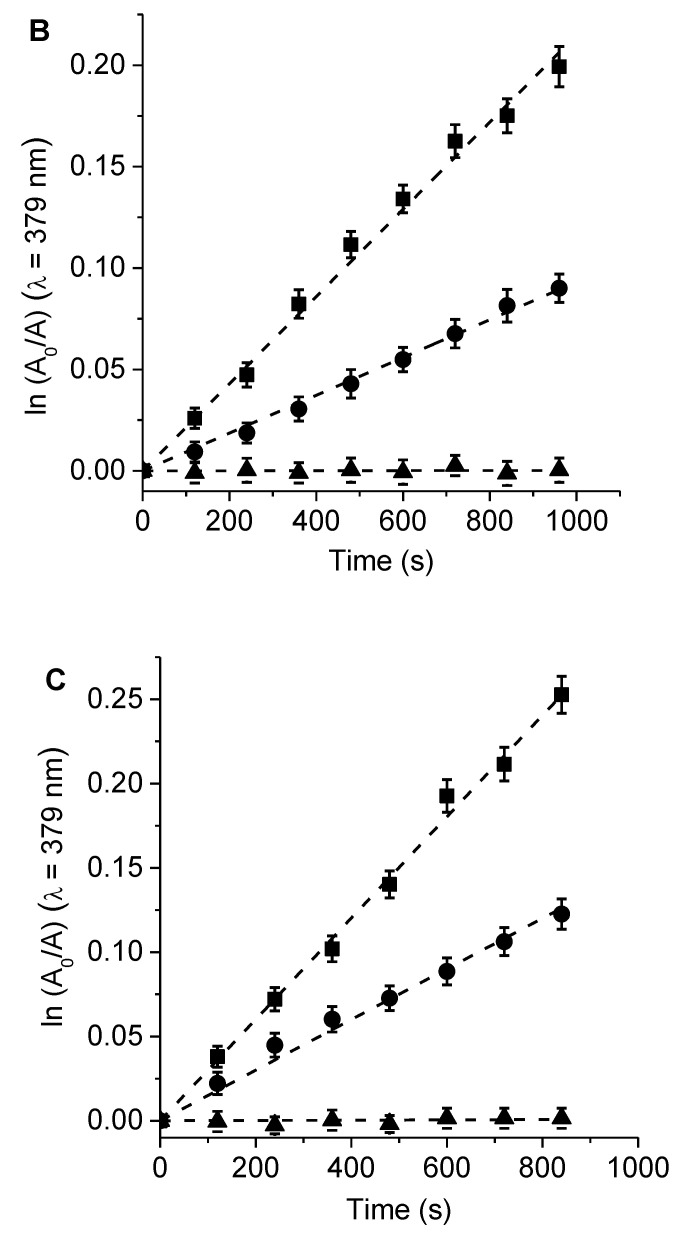
First-order plots for the photo-oxidation of DMA sensitized by porphyrins (**A**) **6**, (**B**) **7,** and (**C**) TMP in DMF (■), DMF/5% water (λ), and DMF/5% water containing 50 mM KI (π); *λ*_irr_ = 517 nm.

The generation of O_2_(^1^Δ_g_) induced by porphyrins **6** and **7** was compared with that of TMP, using DMA as molecular probe in DMF [28]. This anthracene derivative mainly quenches O_2_(^1^Δ_g_) by a chemical reaction [33]. The samples were irradiated at 517 nm under aerobic conditions. The photodegradation of DMA was studied by monitoring its decomposition from the absorption spectra (Figure S3). In all cases, photo-oxidation followed a pseudo-first order kinetic reaction at a wavelength of 379 nm with respect to the DMA concentration (Figure 4). Table 1 presents a summary of the values of the observed rate constant of DMA (*k*_obs_^DMA^). These results were used to determine the quantum yield of O_2_(^1^Δ_g_) production (Φ_Δ_) by comparing the slope obtained for each PS with the value corresponding to the reference, TMP [15]. Similar Φ_Δ_ values were found for porphyrins **6** and **7** in DMF (Table 1). The production of O_2_(^1^Δ_g_) sensitized by these compounds was consistent with previously reported results for structurally related porphyrins [7,14]. Thus, the long lifetime of the triplet excited state of the porphyrins allows for the efficient production of O_2_(^1^Δ_g_) [28]. In addition, the O_2_(^1^Δ_g_) yields of tetrapyrrolic macrocycles can be increased via the formation of zinc complexes [34].

On the other hand, the decomposition of DMA sensitized by **6**, **7**, and TMP was examined in the presence of KI in DMF/5% water. This study was conducted because, as discussed below, KI was used to potential the cytotoxic action of PSs on microbial cells [35,36]. First, the photo-oxidation reaction of DMA was performed in the absence of the inorganic salt in DMF/5% water (Figure 4). In all cases, a decrease in *k*_obs_^DMA^ values was found in this more polar medium (Table 1). This effect may have been due to a reduction in the lifetime of O_2_(^1^Δ_g_) in the presence of water or a change in the reaction rate constant of DMA [37]. After that, the effect of 50 mM KI on the decomposition of DMA induced by these porphyrins was investigated (Figure 4). Under these conditions, the reaction rate of DMA photo-oxidation sensitized by porphyrins **6**, **7**, and TMP was negligible in the presence of this inorganic salt (Table 1). It was previously observed that the addition of KI can act as an antioxidant, promoting the recovery of photo-oxidized fluorophores [38].

### 2.4. Photo-Oxidation of L-Tryptophan (Trp)

The efficacy of porphyrins as photosensitizing agents can be studied using compounds of biological origin, such as amino acids and nucleotides [27,39]. These substrates are potential targets for porphyrin-mediated PDI in microbial cells. Therefore, the amino acid Trp was used to analyze the photodynamic activity of **6**, **7,** and TMP. This amino acid is susceptible to modifications due to the presence of oxidizing agents, and it can be efficiently decomposed by a type I or type II mechanism. Thus, changes in the structure of Trp can cause significant alterations in the physicochemical properties of proteins and subsequently cause cell damage [40].

**Figure 5:**
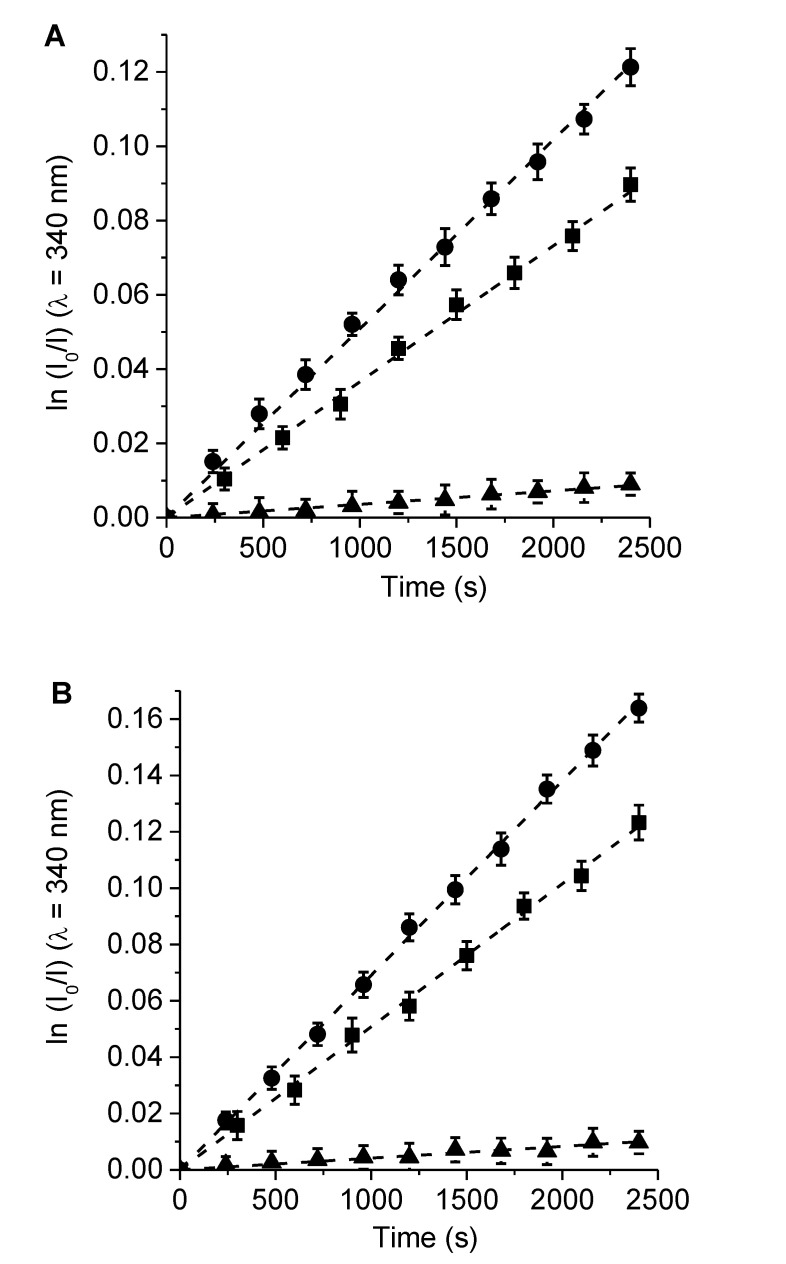

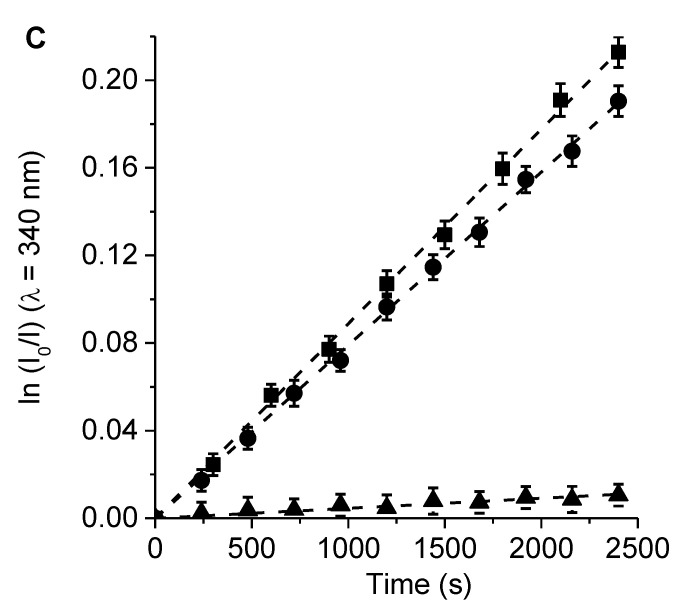
First-order plots for the photo-oxidation of Trp sensitized by porphyrins (**A**) **6**, (**B**) **7,** and (**C**) TMP in DMF (■), λ_irr_ = 517 nm; DMF/10% water (λ), λ_irr_ = 350–800 nm; and DMF/10% water containing 100 mM KI (π), λ_irr_ = 350–800 nm.

The photodecomposition of Trp sensitized by **6**, **7,** and TMP was evaluated by the decrease of fluorescence at 340 nm in DMF (Figure S4). The irradiation of Trp solutions in the presence of PSs was performed under aerobic conditions at 517 nm. As shown in Figure 5, the decomposition of Trp followed first-order kinetics with respect to this amino acid concentration. The observed rate constant of Trp (*k*_obs_^Trp^) values for both porphyrins and the reference were calculated from the plots in Figure 5, and the results are given in Table 2. These results indicated a slightly higher value of *k*_obs_^Trp^ for the reaction photosensitized by porphyrin **7** than that of **6**. On the other hand, the results agreed with the observed O_2_(^1^Δ_g_) production sensitized by these molecules, which indicates that there is a direct correlation between the disappearance of Trp and the production of O_2_(^1^Δ_g_). Both PSs were found to have considerable photodynamic activity in DMF with a significant contribution from the type II mechanism.

Since the photo-oxidation of DMA and Trp was studied in the same solvent (DMF) and source of irradiation (λ_irr_ = 517 nm), the reaction rate constants of Trp induced by these PSs were determined considering a type II mechanism (*k*_r_^Trp^ = *k*_obs_^Trp^ *k*_r_^DMA^/*k*_obs_^DMA^, where *k*_r_^DMA^ = 5 × 10^7^ M^−1^ s^−1^) [41]. Values of *k*_r_^Trp^~1 × 10^7^ M^−1^ s^−1^ were found for the reaction sensitized by these porphyrins (Table 2). These results were similar to that found for the photo-oxidation of Trp mediated by a type II mechanism in DMF [41]. Therefore, a type II mechanism is the main pathway for the photo-oxidation of this amino acid [42].

Furthermore, the effect of the KI addition was studied on the photodecomposition of Trp mediated by **6**, **7,** and TMP in DMF/10% water. These studies were carried out in DMF/10% water to later compare them with the photoinduced iodine generation. To mimic the irradiation conditions used in vitro, the samples were exposed to visible light (350–800 nm). Thus, the oxidation reaction of Trp was first investigated in this medium, as shown in Figure 5. The values of *k*_obs_^Trp^ are summarized in Table 2. A higher value of *k*_obs_^Trp^ was also found for **7** with respect to **6**. In the presence of KI (100 mM), the values of *k*_obs_^Trp^ decreased by more than one order of magnitude (~16 times) with respect to the same experiment in the absence of the inorganic salt. This effect could be attributed to the reaction of O_2_(^1^Δ_g_) with iodide anions to generate iodine [35]. This process competed with the photo0oxidation of Trp, producing a decrease in the decomposition rate of the amino acid. In addition, a control test was performed to evaluate the stability of Trp in the presence of 20 µM Lugol (aqueous solution of I_2_ + I^−^) in DMF/10% water, with the solution kept in the dark (Figure S5). The results confirmed that Trp was not affected by the presence of iodine in the medium.

### 2.5. Photoinduced Iodine Generation

**Figure 6:**
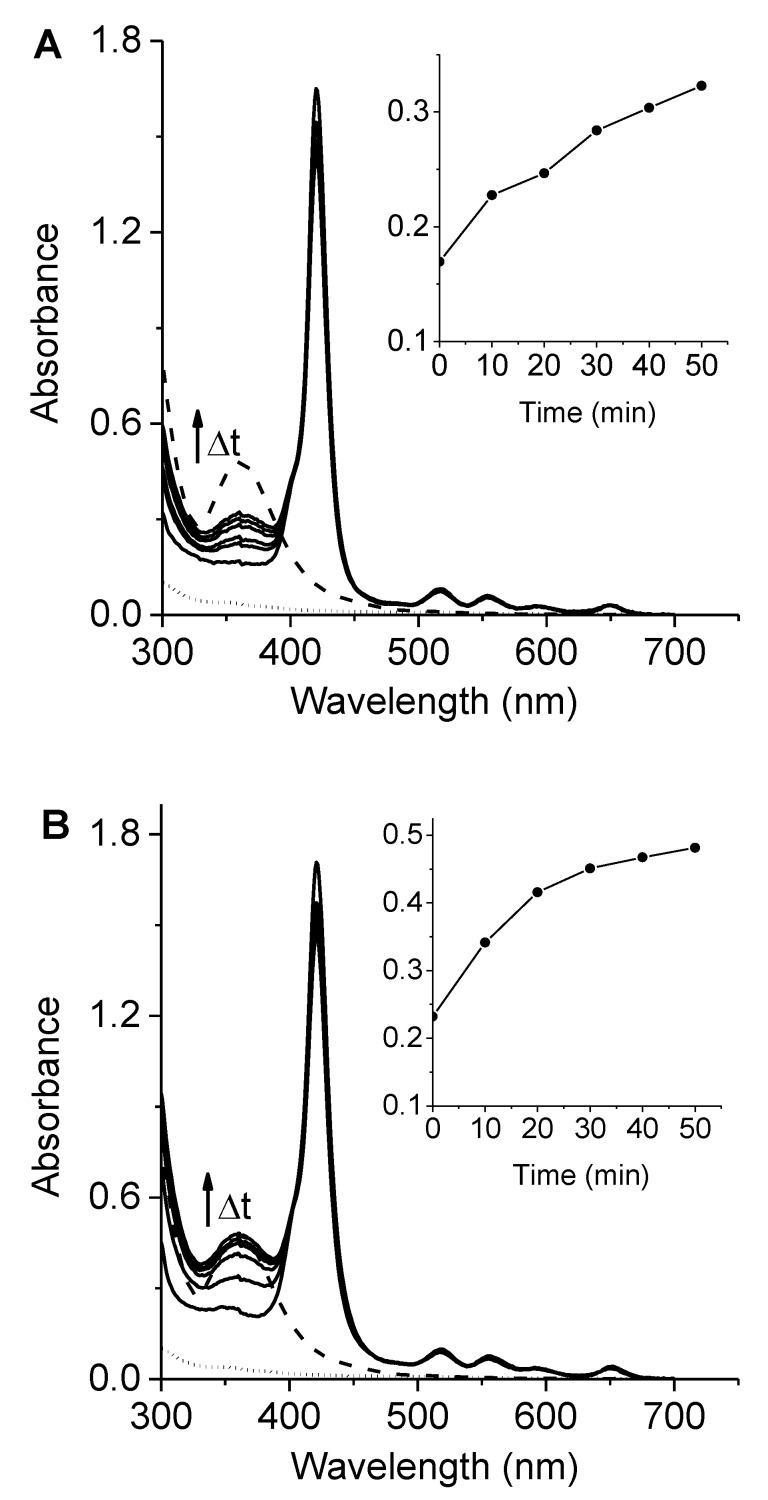
Absorption spectra of (**A**) **6** and (**B**) **7** containing 100 mM KI in DMF/10% after different irradiation times (0, 10, 20, 30, 40, and 50 min; solid lines) with visible light. Inset: changes in absorbance at 360 nm after different irradiation times. The dashed and dotted lines represent Lugol’s solution (20 μM) and KI (100 mM), respectively, irradiated with visible light for 50 min.

To determine the generation of iodine in the system, the UV–visible absorption spectra of porphyrin **6** or **7** in DMF/10% water containing 100 mM KI were recorded after different irradiation times with visible light (350–800 nm). As shown in Figure 6, a new band appeared at 360 nm and was observed to enlarge with the irradiation time. This band was assigned to the formation of iodine in the solution [43,44]. Furthermore, the spectra were compared with those of a positive control, diluted 20 μM Lugol in DMF/10% water. The absorbance of iodine at 360 nm was registered at different irradiation times, and the results are shown in the inset of Figure 6. The absorbance at this wavelength gradually increased with the exposure time for both porphyrins in the presence of KI. As can be observed, porphyrin **7** was a more effective iodine generator than **6**. These results correlated with the production of O_2_(^1^Δ_g_) of these porphyrins. A similar tendency was previously found using cationic pyrrolidine/pyrroline-substituted porphyrins as PSs in the presence of KI [45]. It was demonstrated that the reaction of iodide anions and O_2_(^1^Δ_g_) resulted in the production of triiodide anions (I_3_^−^) in aqueous media [35]. In this process, hydrogen peroxide (H_2_O_2_) can be formed. However, this intermediate can further react with iodide anions to generate I_3_^‒^ (Scheme S3) [46,47].

### 2.6. PDI of Microbial Cells

**Figure 7:**
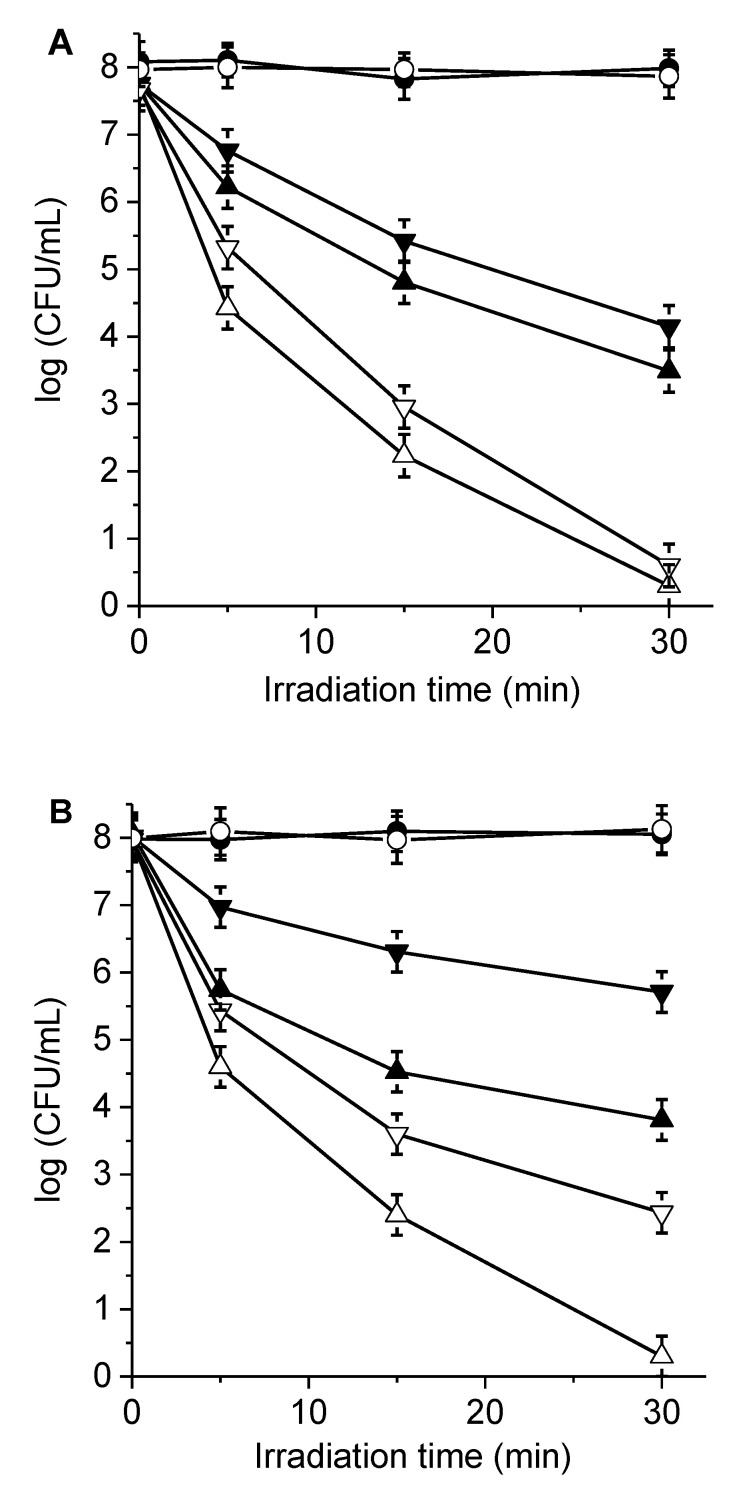

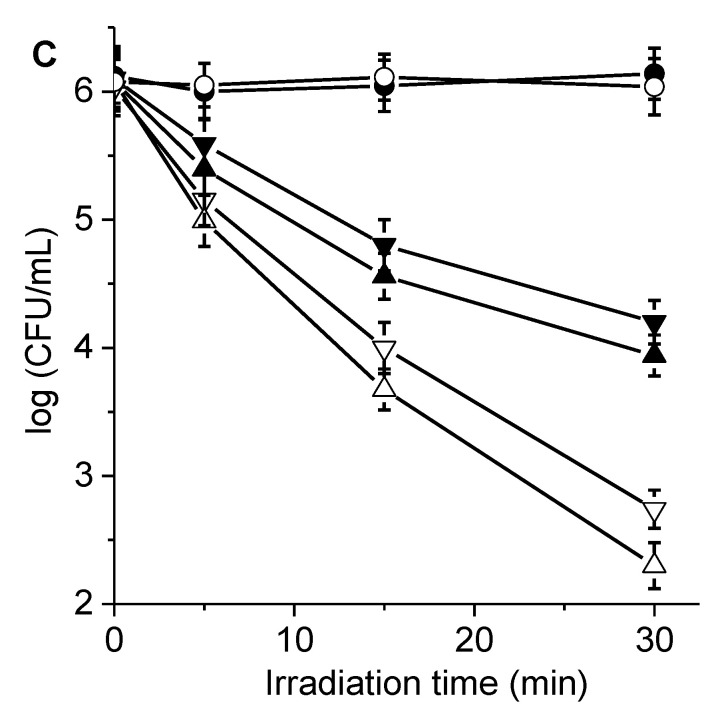
Survival curves of (**A**) *S. aureus* (~10^8^ CFU/mL) treated with 1 µM porphyrin, (**B**) *E. coli* (~10^8^ CFU/mL) treated with 10 µM porphyrin, and (**C**) *C. albicans* (~10^6^ CFU/mL) treated with 5 µM porphyrin for 30 min in the dark and irradiated with visible light for different periods. Additionally shown are porphyrin **6** in the absence (θ) and in the presence of KI (σ) and porphyrin **7** in the absence (π) and in the presence of KI (△). *S. aureus* cells were incubated with 50 mM KI, and *E. coli* and *C. albicans* were incubated with 100 mM KI. Additionally shown are an irradiated control culture without porphyrin (λ) and irradiated control culture treated with KI without porphyrin (○).

Photodynamic activity sensitized by porphyrins **6** and **7** were evaluated in *S. aureus*, *E. coli,* and *C. albicans* cell suspensions. These microbial strains were selected because they represent possible pathogens with different degrees of complexity in the cell wall [17,18,19]. Microorganism cultures suspended in PBS were treated with each porphyrin for 30 min at 37 °C in the dark. Subsequently, the cells were exposed to visible light for different times (5, 15, and 30 min, which represent 27, 81, and 162 J/cm^2^, respectively). *S. aureus*, *E. coli*, and *C. albicans* were incubated with 1.0, 10.0, and 5.0 μM porphyrin, respectively. At these concentrations, the porphyrins were not toxic to the microbial cells for 30 min of incubation in the dark (Figure S6). Moreover, the viability of the microorganisms was not affected by cell irradiation without porphyrin (Figure 7). Therefore, the PDI of cultures observed after irradiation of the cells treated with porphyrins was caused by the photodynamic activity produced by these compounds.

The survival of microbial cells after different PDI treatments is illustrated in Figure 7. Cell viability depended on both the porphyrin derivatives and the times of exposure to visible light. After 30 min of irradiation, the photoinactivation of 3.8 and 4.5 log decreases in bacterial survival were found in *S. aureus* cells (~10^8^ CFU/mL) treated with 1.0 μM porphyrins **6** and **7**, respectively (Figure 7A). The photokilling activity induced by both porphyrins was similar in this Gram-positive bacterium, although slightly higher (~0.7 log) for porphyrin **7** than **6**. The reduction in viability produced by **7** represented a value greater than 99.996% of cell inactivation. Furthermore, porphyrin **7** showed a 3.2 log reduction (99.94%) photodynamic effect after 15 min of irradiation.

In *E. coli*, cell suspensions (10^8^ CFU/mL) incubated with 10.0 μM porphyrin **7** and irradiated for 15 min produced a 3.5 log decrease in viability, and after 30 min of irradiation, they showed a photosensitizing inactivation of 4.5 log units (Figure 7B). The last photokilling result represented a reduction of cell survival greater than 99.996%. In the last condition, the photoinactivation mediated by porphyrin **6** was considerably less than **7**, producing a reduction of 2.2 log. The difference in the photoinactivating capacity between porphyrins **6** and **7** was more significant in this Gram-negative bacterium, where the presence of the two precursor groups of positive charges in porphyrin **7** allowed for better interactions with the cell walls of bacteria. Similar results were previously found for *E. coli* incubated with 5,15-di(4-pentafluorophenyl)-10,20-di (4-(3-*N*,*N*-dimethylaminopropoxy)phenyl)porphyrin (F_10_APP), which is related to porphyrin **7** because it is also substituted by two basic amino groups [7].

PDI studies were also performed in *C. albicans* cell suspensions (10^6^ CFU/mL), which were incubated with 5.0 μM porphyrin. As shown in Figure 7C, the photodynamic action induced for both porphyrins was similar in yeast cells. After 15 min of irradiation, we detected a survival decrease of ~1.5 log in the *C. albicans*. The photokilling reached 2.0 log (99%) when yeast cells incubated with porphyrin **7** were irradiated for 30 min. This PDI of *C. albicans* cells was comparable to that reported for the same yeast strain sensitized by F_10_APP [7].

On the other hand, the PDI of microbes sensitized by porphyrins **6** and **7** was investigated in the presence of KI. *S. aureus* cells were incubated with 50 mM KI, and *E. coli* and *C. albicans* were incubated with 100 mM KI. These concentrations of KI were selected in consideration of previous reports for the potentiation of PDI [33,35]. This inorganic salt was not found to be toxic for microorganisms exposed to irradiation for 30 min (Figure 7). In *S. aureus*, the combined treatment of the photodynamic action and the addition of KI produced an increase in the photoinactivation of about 2.0 log (Figure 7A). Under these conditions, the nearly complete eradication of the bacteria was achieved after 30 min of irradiation. Furthermore, an enhancement by KI was found in the photokilling of *E. coli* compared to the inactivation alone, mainly at the longer irradiation times (15 and 30 min). After 30 min of irradiation, addition of KI produced a 2.5 log increase in bacterial photoinactivation for the Gram-negative bacterium sensitized by porphyrin **7**. Therefore, the combined therapy using **7** and KI produced a decrease greater than 7 log in cell viability. Moreover, the photoinactivation of *C. albicans* was improved by the addition of KI (Figure 7C) with respect to PDI in the absence of the inorganic salt. After 30 min of irradiation, a potentiated photoinactivation of 1.8 log was observed for porphyrin **7**, reaching a total decrease of 3.8 log (99.98%). A similar effect of iodide anions was found for porphyrin **6**, although survival was reduced by 3.2 log in this case. It was reported that the efficacy of PDI mediated by methylene blue was considerably increased in *S. aureus* and *E. coli* after the addition of KI [48]. Additionally, the PDI of bacteria and yeast cells sensitized by cationic fullerenes was potentiated by iodide anions [33,49,50]. Even KI was able to enhance the photokilling of microorganisms induced by BODIPYs, which have a low production of O_2_(^1^Δ_g_) [35]. Potentiation by iodide anions was also used to improve the PDI of microorganisms sensitized by several monocationic porphyrins and anionic porphyrins [45,51,52]. Therefore, the interaction of the ROS and KI due to the light activation of PSs produces biocidal I_2_ or I_3_^-^ that improve bacterial inactivation [53]. Consequently, this alternative pathway of cytotoxicity allows for the enhancement of the PDI of microbial cells.

## 3. Materials and Methods

Supplies, instrumentation, and computational details are described in the Supporting Information.

### 3.1. Synthesis of Meso-Substituted Porphyrins

5-(4-Aminophenyl)-10,15,20-tris(4-methylphenyl)porphyrin (**3**) (Scheme S1), 5,10-di(4-aminophenyl)-15,20-di(4-methylphenyl)porphyrin (**5**) (Scheme S2), and TMP were synthesized as described in the literature [21,22,23,34].

5-((4-(4-(3-(*N*,*N*-dimetylamino)propoxy)benzylidene)amino)phenyl)-10,15,20-tris(4-metylphenyl)porphyrin (**6**): A solution of **3** (31 mg; 0.046 mmol) and 4-(3-(*N*,*N*-dimethylamino)propoxy)benzaldehyde (50 μL; 0.25 mmol) in 20 mL of dry toluene containing molecular sieves (4 Å) was refluxed under an argon atmosphere for 30 h until the complete disappearance of porphyrin **3**, as indicated by TLC (silica gel). The mixture was filtered and washed with DCM/5% methanol/1% TEA. The solvent was removed under reduced pressure, and the product was purified with a flash chromatography column (silica gel, DCM/5% methanol/1% TEA) to DCM/30% methanol/5% TEA gradient. Subsequent crystallization with DCM/petroleum ether (1:3) containing 1% TEA afforded 34 mg (86%) of **6** as a purple solid. ^1^HNMR (CDCl_3_, TMS) δ (ppm): −2.78 (2H, pyrrole NH), 2.04 (m, 2H, -CH_2_-), 2.34 (s, 6H, -N(CH_3_)_2_), 2.46 (t, 2H, *J* = 7.1 Hz, -CH_2_N), 2.69 (s, 9H, Ar-CH_3_), 4.02 (t, 2H, *J* = 7.0 Hz, O-CH_2_-), 7.00 (d, 2H, 3′,5’-Ar, *J* = 7.9 Hz), 7.55 (d, 6H, 3,5-Ar, *J* = 8.0 Hz), 7.70 (d, 2H, 2,6-Ar, *J* = 8.0 Hz), 8.01 (d, 2H, 2´,6´-Ar, *J* = 7.9 Hz), 8.10 (d, 6H, 2,6-Ar, *J* = 8.0 Hz), 8.22 (d, 2H, 3,5-Ar, *J* = 8,0 Hz), 8.76 (s, 1H, -N=CH-), 8.83–8.90 (m, 8H, β-pyrrole). ^13^CNMR (100 MHz, CDCl_3_) δ (ppm) 21.5, 29.2, 45.5, 58.3, 68.4, 119.6, 114.8, 122.8, 127.9, 128.2, 129.7, 130.9, 131.7, 134.8, 137.1, 140.2, 140.9, 147.5, 150.8, 160.9, 161.5. HRMS-ESI (m/z): 861.4288 (861.4281 calculated for [M + H]^+^, M = C_59_H_52_N_6_O). UV–visible (DMF) λ_max_ (nm) (log ε): 422 (5.63), 518 (4.31), 556 (4.24), 598 (3.94), 650 (3.92). Fluorescence emission (DMF) λ_max_ (nm): 654, 720. The same procedure was used to obtain 5,10-di((4-(4-(3-*N*,*N*-dimetylaminopropoxy)phenyl) imino)metylphenyl)-15,20-di(4-metylphenyl) porphyrin (**7**), starting from **5** (23 mg; 0.034 mmol) and 4-(3-(*N*,*N*-dimethylamino)propoxy)benzaldehyde (50 μL; 0.25 mmol). After purification, porphyrin 7 was obtained in a 83% yield (30 mg). ^1^HNMR (CDCl_3_, TMS) δ (ppm): −2.75 (2H, pyrrole NH); 2.06 (m, 4H, -CH_2_-), 2.35 (s, 12H, -N(CH_3_)_2_), 2.47 (t, 4H, *J* = 7,1 Hz, -CH_2_N); 2,69 (s, 6H, Ar-CH_3_), 4,09 (t, 4H, *J* = 7,0 Hz, O-CH_2_-), 7.01 (d, 4H, 3´,5´-Ar, *J* = 7.9 Hz), 7.55 (d, 4H, 3,5-Ar, *J* = 8.0 Hz), 7.72 (d, 4H, 2,6-Ar, *J* = 8,0 Hz), 7.98 (d, 4H, 2´,6´-Ar, *J* = 7.9 Hz), 8.09 (d, 4H, 2,6-Ar, *J* = 8,0 Hz), 8.21 (d, 4H, 3,5-Ar, *J* = 8,0 Hz), 8,73 (s, 2H, -N=CH-), 8.82–8.94 (m, 8H, β-pyrrole). ^13^CNMR (100 MHz, CDCl_3_) δ (ppm) 21.7, 28.9, 46.1, 57.8, 68.1, 119.2, 114.3, 123.1, 127.6, 128.0, 129.2, 131.1, 131.9, 135.1, 137.5, 140.1, 141.2, 147.6, 150.2, 160.3, 161.3. HRMS-ESI (m/z) 1051.5388 [M + H]^+^ (1050.5309 calculated for C_70_H_66_N_8_O_2_). UV–visible (DMF) λ_max_ (nm) (log ε): 422 (5.62), 518 (4.36), 556 (4.24), 597 (3.90), 652 (3.88). Fluorescence emission (DMF) λ_max_ (nm): 656, 721.

### 3.2. Spectroscopic Studies

A quartz cell (1 cm path length) was used to acquire UV–visible absorption and fluorescence spectra in DMF. Samples were excited at 517 nm; the absorbance was in all cases ~0.05, and the emission spectra were integrated in the range 600–800 nm [7,14]. The energy of the singlet state (*E*s) was calculated from the intersection of absorption and normalized fluorescence curves. The fluorescence quantum yield (Φ_F_) of the porphyrins was calculated from the area below the corrected emission spectrum using TMP as a reference (Φ_F_ = 0.14) in the same solvent [15].

### 3.3. Photo-Oxidation of DMA

A solution of 9,10-dimethylanthracene (DMA; 35 μM) and porphyrin (A = 0.1 at 517 nm) were prepared in 2 mL of DMF, and the samples were irradiated with light at λ_irr_ = 517 nm. The photo-oxidation rate of DMA was analyzed by determining the decrease in absorbance at λ_max_ = 379 nm. The values of *k*_obs_^DMA^ were calculated by a linear least-squares fit of the pseudo-first order kinetic plots of ln(A_0_/A) vs. time. The quantum yields of O_2_(^1^Δ_g_) production (Φ_Δ_) were determined comparing the *k*_obs_^DMA^ for the corresponding PS with that for TMP, which was used as a reference (Φ_Δ_ = 0.65) [8]. In addition, the photo-oxidation of DMA by porphyrins was evaluated in DMF/5% water and in the presence of 50 mM KI in the same medium [35]. The inorganic salt was added from a stock solution (1.0 M) in water.

### 3.4. Photo-Oxidation of Trp

Solutions of Trp (25 µM) and porphyrin in 2 mL of DMF (A = 0.1 at 517 nm) were treated as described above for the photodecomposition of DMA. The photo-oxidation of Trp was studied by exciting the samples at λ_exc_ = 290 nm and following the decrease of the fluorescence intensity at λ_em_ = 340 nm [35]. The values of *k*_obs_^Trp^ were determined by a linear least-squares fit of semi-logarithmic plots of ln(I_0_/I) vs. time. Experiments in DMF/10% water and in the presence of 100 mM KI were performed as described above for DMA, though we irradiated the samples with visible light (350–800 nm).

### 3.5. Generation of Iodine

Solutions of the porphyrins (5 µM) in DMF/10% water (2 mL) were studied in the presence of 100 mM KI using a stock of the salt prepared in water (1.0 M). The experiment was carried out as described above for the photodecomposition of Trp, irradiating the samples with visible light (350–800 nm). The generation of iodine was studied by UV–visible spectroscopy following the absorbance at 360 nm after different irradiation times (0–50 min) [36,50]. A solution of Lugol was used as a positive control.

### 3.6. Photoinactivation of Microorganisms

The growth conditions of the microbial strains and the statistical analysis are detailed in the Supporting Information. Microbial cell suspensions in PBS (2 mL; ~10^8^ CFU/mL for bacteria and ∼10^6^ CFU/mL for yeast) were incubated with porphyrin (1.0 μM for *S. aureus*, 10.0 μM for *E. coli*, and 5.0 μM for *C. albicans*) in culture tubes (Pyrex, 13 mm × 100 mm) for 30 min in the dark at 37 °C [7,15]. Porphyrins were added from 0.5 mM stock solutions in DMF. After that, 200 µL of the cell suspensions were transferred to each well of 96-well microtiter plate. The cultures were irradiated for different times (5, 15, and 30 min) with visible light at 350–800 nm and a fluence rate of 90 mW/cm^2^ [54]. A similar procedure was used to determine the PDI in the presence of KI (50 mM for *S. aureus* and 100 mM for *E. coli* and *C. albicans*). KI was added from 1.0 M aqueous stock solution. Microbial cells were incubated with KI for 10 min in the dark at 37 °C, and then cells were treated with porphyrin as described above. Viable cells were quantified as mentioned above.

## 4. Conclusions

Two new conjugates of porphyrin–Schiff base conjugates **6** and **7** were synthesized from the corresponding tetrapyrrolic macrocycle functionalized with amino groups in an AB_3_- and AABB-type structures, respectively. These compounds can be protonated at physiological pH, facilitating the interaction of PSs with the cell walls of microorganisms. The presence of the substituents attached through the Schiff bases in porphyrins **6** and **7** did not affect the spectroscopic characteristics of absorption and fluorescence. Photodynamic studies in a homogeneous medium showed that these compounds exhibited the considerable production of O_2_(^1^Δ_g_). Furthermore, these porphyrins induced the photodecomposition of Trp with a significant contribution of type II photoprocess. In the presence of iodide anions, both DMA and Trp photo-oxidations were strongly quenched by reaction with O_2_(^1^Δ_g_) to produce iodine. PDI studies indicated that porphyrin **7** was more effective than **6** at inactivating microorganisms. This difference was more significant in Gram-negative bacterium, where the cell wall plays a preponderant role. In contrast, the photoinactivating effectiveness was slightly higher for **7** than **6** in the Gram-positive bacterium and yeast. The results in bacteria showed that porphyrin **7** can be considered an antimicrobial agent since it is capable of reducing more than 3 log in cell viability. Furthermore, the photoinactivation of these microorganisms was considerably improved by the addition of KI. The PDI treatment combined with the potentiation by iodide anions allowed us to achieve the complete eradication of bacteria and a reduction of more than 3 log in *C. albicans*. Therefore, porphyrins that contain basic aliphatic amino groups can acquire positive charges in biological media, increasing the interaction with the cell walls of microorganisms and the photoinactivating capacity of pathogens.

## Figures and Tables

**Table 1:** Kinetic parameters for the photo-oxidation reaction of DMA (*k*_obs_^DMA^) sensitized by **6**, **7**, and TMP, as well as quantum yields of O_2_(^1^Δ_g_) production (Φ_Δ_).

Porphyrin	*k*_obs_^DMA^ (s^−1^) ^a^	*k*_obs_^DMA^ (s^−1^) ^b^	*k*_obs_^DMA^ (s^−1^) ^c^	Φ_Δ_ ^a^
**6**	(1.96 ± 0.05) × 10^−4^	(0.85 ± 0.03) × 10^−4^	ND	0.42 ± 0.02
**7**	(2.15 ± 0.06) × 10^−4^	(0.93 ± 0.04) × 10^−4^	ND	0.46 ± 0.02
TMP	(3.01 ± 0.08) × 10^−4^	(1.50 ± 0.06) × 10^−4^	ND	0.65 ± 0.03 ^d^

^a^ DMF; ^b^ DMF/5% water; ^c^ DMF/5% water in the presence of 50 mM KI; ^d^ from [15]; ND: not detected.

**Table 2:** Kinetic parameters for the photo-oxidation of Trp (k_obs_^Trp^) sensitized by **6**, **7,** and TMP.

Porphyrin	*k*_obs_^Trp^ (s^−1^) ^a^	*k*_r_^Trp^ (M^−1^ s^−1^)	*k*_obs_^Trp^ (s^−1^) ^b^	*k*_obs_^Trp^ (s^−1^) ^c^
**6**	(3.66 ± 0.06) × 10^−5^	(9.33 ± 0.09) × 10^6^	(5.12 ± 0.05) × 10^−5^	(3.60 ± 0.08) × 10^−6^
**7**	(5.08 ± 0.05) × 10^−5^	(1.18 ± 0.01) × 10^7^	(6.90 ± 0.06) × 10^−5^	(4.15 ± 0.09) × 10^−6^
TMP	(8.88 ± 0.08) × 10^−5^	(1.47 ± 0.01) × 10^7^	(7.89 ± 0.07) × 10^−5^	(4.57 ± 0.08) × 10^−6^

^a^ DMF, λ_irr_ = 517 nm; ^b^ DMF/10% water λ_irr_ = 350–800 nm; ^c^ DMF/10% water in the presence of 100 mM KI, λ_irr_ = 350–800 nm.

## Data Availability

Data supporting the findings of this investigation are available within the article and its Supplementary Materials.

## Supplementary Materials

The following, containing a detailed description of the materials, instrumentation, computational details, growth conditions of microbial strains, and statistical analysis, are available online: Scheme S1: synthesis of porphyrin **3**, Scheme S2: synthesis of porphyrin **5**, Scheme S3: reaction of O_2_(^1^Δ_g_) with iodide anions in aqueous media, Scheme S4: model of irradiation systems for (A) steady state photolysis and (B) PDI, Figure S1: optimized structure of non-charged **6** and **7** and protonated analogs **6^+^** and **7^2+^** calculated for ground state by using DFT at the B3LYP/6-31G(d) level and red arrows indicate the calculated relative magnitude and orientation of permanent dipole moment (*μ*), Figure S2: Highest occupied molecular orbital (HOMO) and lowest unoccupied molecular orbital (LUMO) of charged porphyrins **6**^+^ (up) and **7**^2+^ (down). All calculations were performed by DFT at the CAM-B3LYP/6–31 + G(d) level using Gaussian 09, Figure S3: absorption spectral changes during the photooxidation of DMA sensitized by **6** in DMF at different irradiation times (Δt = 120 s), λ_irr_ = 517 nm, Figure S4: fluorescence spectral (λ_exc_ = 290 nm) changes during the photodecomposition of Trp sensitized by **6** in DMF at different irradiation times (Δt = 300 s), λ_irr_ = 517 nm, Figure S5: first-order plots for the stability of Trp in the presence of Lugol (20 μM) in DMF/10% water in the dark, Figure S6: survival curves of (A) *S. aureus* (∼10^8^ CFU/mL)*,* (B) *E. coli* (∼10^8^ CFU/mL) and (C) *C. albicans* (∼10^6^ CFU/mL) treated with porphyrin **6** and **7** for different times in the dark at 37 °C.

## Sample Availability

Samples of the compounds are not available from the authors.
