# Supplementary material for: Porphyrin–Schiff Base Conjugates Bearing Basic Amino Groups as Antimicrobial Phototherapeutic Agents
Source: Molecules. 2021 Sep 28;26(19):5877. doi: 10.3390/molecules26195877 (PMC8510454; doi:10.3390/molecules26195877)
Supplement: Supplementary file 1 [file molecules-26-05877-s001.zip › molecules-1377940-SI.pdf]

Supporting Information

Porphyrin-Schiff base conjugates bearing basic amino groups as antimicrobial phototherapeutic agents

M. Eugenia Pérez[1], Javier E. Durantini[2], Eugenia Reynoso[1], María G. Alvarez[1], María E. Milanesio[1]* and Edgardo N. Durantini[1]*

[1] IDAS-CONICET, Departamento de Química, Facultad de Ciencias Exactas, Físico-Químicas y Naturales, Universidad Nacional de Río Cuarto, Ruta Nacional 36 Km 601, X5804BYA Río Cuarto, Córdoba, Argentina.

[2] IITEMA Departamento de Química, Facultad de Ciencias Exactas, Físico-Químicas y Naturales, Universidad Nacional de Río Cuarto, Ruta Nacional 36 Km 601, X5804BYA Río Cuarto, Córdoba, Argentina.

Table of Contents

1. Materials Page S2

2. Instrumentation Page S2

3. Computational details Page S3

4. Growth conditions of microbial strains Page S3

5. Statistical analysis Page S4

6. Supporting schemes and figures Page S4

7. References Page S8

* Correspondence: mmilanesio@exa.unrc.edu.ar (M.E.M.); edurantini@exa.unrc.edu.ar (E.N.D.)

1. Materials

Chemicals were obtained from Sigma-Aldrich (Milwaukee, WI, USA). These compounds were used without further purification. Organic solvents (GR grade) from Merck (Darmstadt, Germany) were distilled and maintained on molecular sieves. Ultrapure water was obtained from a Labconco (Kansas City, MO, USA) equipment model 90901-01. Silica gel thin-layer chromatography (TLC) plates (250 microns) were acquired from Analtech (Newark, DE, USA) and silica gel 60 (0.040-0.063 mm, 230-400 mesh) from Merck (Darmstadt, Germany). Tryptic soy (TS) broth and agar from Britania (Buenos Aires, Argentina) were used in microbial cultures. Microtiter plates (96-well) were acquired to Deltalab (Barcelona, Spain).

2. Instrumentation

Proton nuclear magnetic resonance spectra were performed on a FT-NMR Bruker Advance DPX400 at 400 MHz (Bruker BioSpin, Rheinstetten, Germany). Mass spectra were recorded on a Bruker micrOTOF-QII (Bruker Daltonics, Billerica, MA, USA) equipped with an ESI source (ESI-MS). IR spectra were recorded on a Bruker Tensor 27 FT-IR (Ettlingen, Germany). Absorption and fluorescence spectra were carried out on a Shimadzu UV-2401PC spectrometer (Shimadzu Corporation, Tokyo, Japan) and on a Spex FluoroMax spectrofluorometer (Horiba Jobin Yvon Inc, Edison, NJ, USA), respectively. Spectroscopic determinations were performed in a quartz cell of 1 cm path length at room temperature. A Radiometer Laser Mate-Q (Coherent, Santa Clara, CA, USA) was used to determinate the light fluence rates. Steady state photolysis in solution were performed with a Cole-Parmer illuminator 41720-series (150 W halogen lamp, Cole-Parmer, Vernon Hills, IL, USA) in combination with a high intensity grating monochromator (Photon Technology Instrument, Birmingham, NJ, USA). This arrangement produces a light fluence rate of 0.84 mW/cm^2 at 517 ± 6 nm. Cell suspensions were irradiated with a Novamat 130 AF (Braun Photo Technik, Nürnberg, Germany) slide projector containing with a 150 W lamp. A 2.5 cm glass cuvette filled with water without circulation was used to remove the heat from the lamp. A wavelength range between 350 and

800 nm was selected by optical filters. Emission spectrum of the light source was previously reported [1]. The projector was placed vertically with the light beam focused on the 96-well microtiter plate lid, producing a fluence rate of 90 mW/cm^2 [2].

3. Computational details

Computational modeling employing density functional theory (DFT) was performed using Gaussian 09 package (Gaussian, Wallingford, CT) with the CAM-B3LYP functional coupled with the 6-31G(d) basis set [3]. This hybrid exchange correlation functional is the long-range corrected version of B3LYP [4]. Geometries for all structures were fully optimized, and conformational searches were achieved to locate the minimum-energy conformers of all of the structures. As an initial step, a large number of geometries were generated through the conformational search modules of Spartan'14 (Wavefunction, Inc., Irvine.) using MMFF force field and then subjected to PM6 optimization. Then all the structures were re-optimized at the CAM-B3LYP/6- 31 G(d) levels of theory. Molecular electrostatic potential (ESP) surfaces of the optimized structures were visualized using GaussView Software Version 6.0, with an iso value of 0.004 e/au^3. Color maps for potential surfaces were chosen to yield maximum contrast of a given net charge and the relative locations of partial positive (blue) and negative (red) charge. Geometry optimized structures, dipolar moment vectors and molecular orbitals were visualized with Avogadro Software, version 1.2.0 [5].

4. Growth conditions of microbial strains

The microorganisms assayed in this study were *S. aureus* (ATCC 25923), *E. coli* (EC7) and *C. albicans* (PC31), which were previously identified and characterized [6]. These strains were grown under aerobiosis overnight at 37 ºC in 4 mL tryptic soy broth for bacteria or Sabouraud broth for yeast. Cultivation of microorganisms and handling of cell cultures to obtain ~10^8 colony forming units (CFU)/mL for bacteria and ~10^6 CFU/mL for yeast in phosphate-buffered saline (PBS, pH = 7.4) were achieved as reported [2,6]. Viable microbial cells were quantified by the spread plate

technique using serial dilutions 10-fold in PBS. Each sample was streaked on tryptic soy (bacteria) or Sabouraud (yeast) agar plates in triplicate. The formation of colonies was counted after incubation of 24 h for bacteria and 48 h for yeast at 37 °C in the dark.

5. Statistical analysis

Each value represents the mean of three separate experiments and the error bar denotes the standard deviation. Microorganism controls were achieved using irradiated cultures without porphyrin and in the presence of PS in the dark. Differences between means were confirmed for significance by one-way ANOVA. Values were considered statistically significant according a confidence level of 95% ($p<0.05$).

6. Supporting schemes and figures

5-(4-Aminophenyl)-10,15,20-tris(4-methylphenyl)porphyrin (**3**) and 5,10-di(4-aminophenyl)-15,20-di(4-methylphenyl)porphyrin (**5**) were synthesized as reported [7-9].

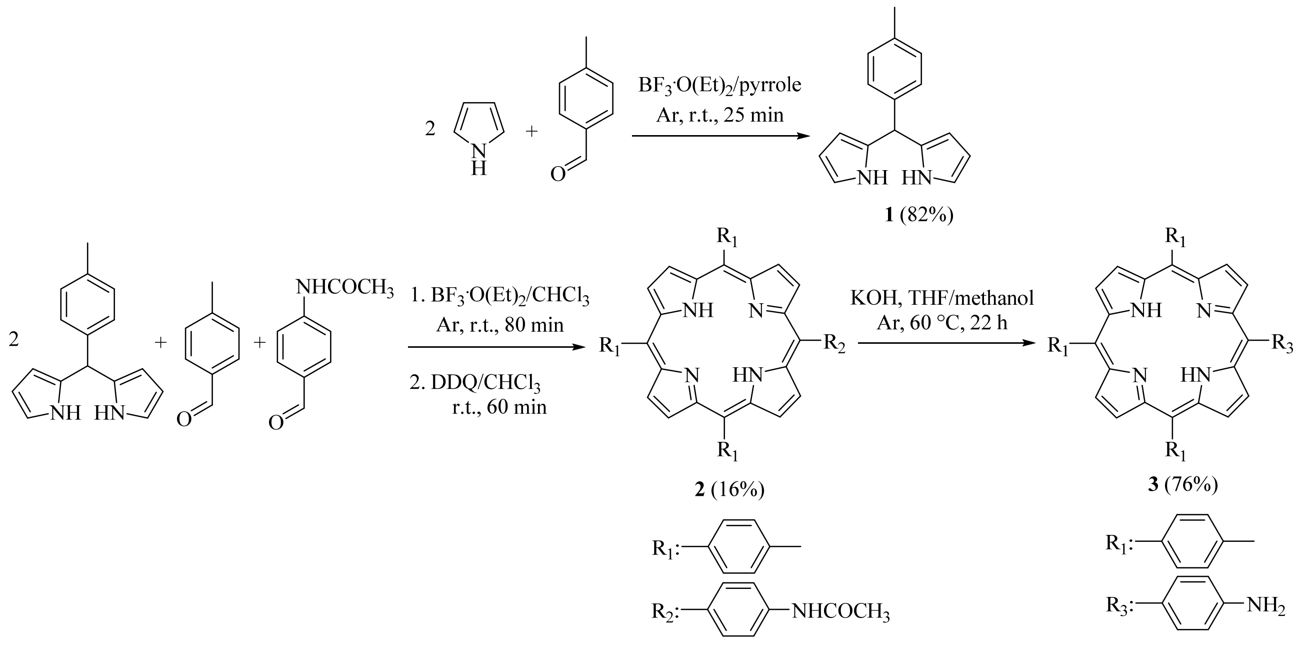

Scheme S1. Synthesis of porphyrin **3**.

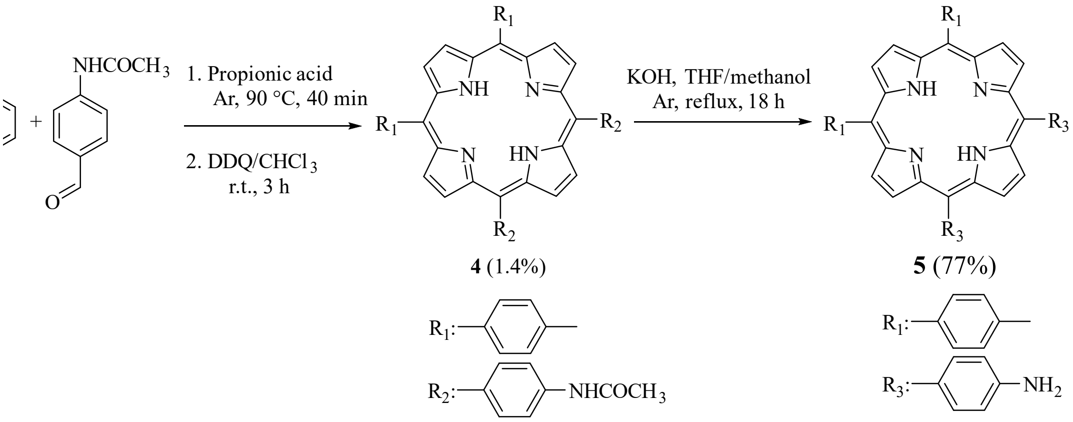

Scheme S2. Synthesis of porphyrin **5**.

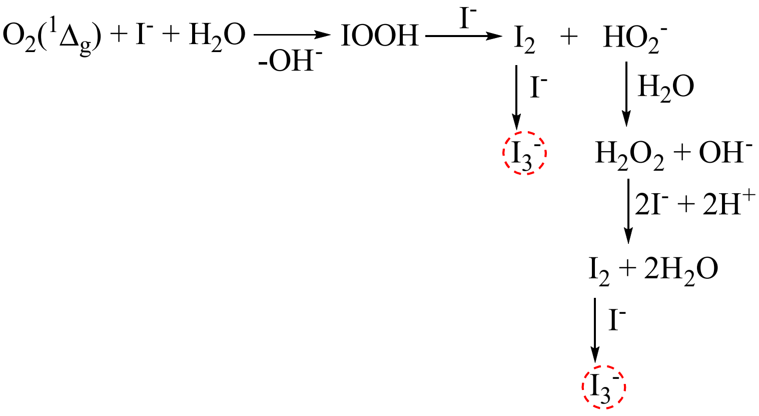

Scheme S3. Reaction of $O_2(^1\Delta_g)$ with iodide anions in aqueous media [6,10].

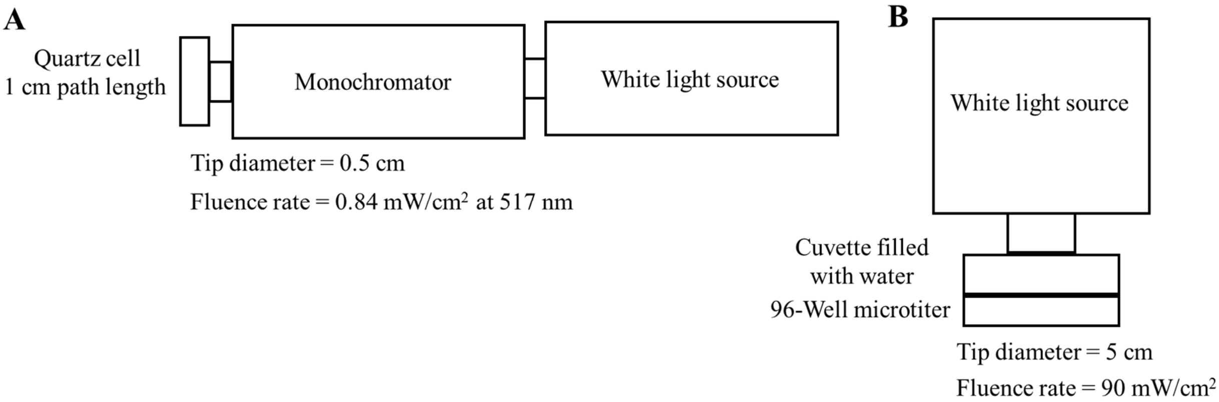

Scheme S4. Model of irradiation systems for (A) steady state photolysis and (B) PDI.

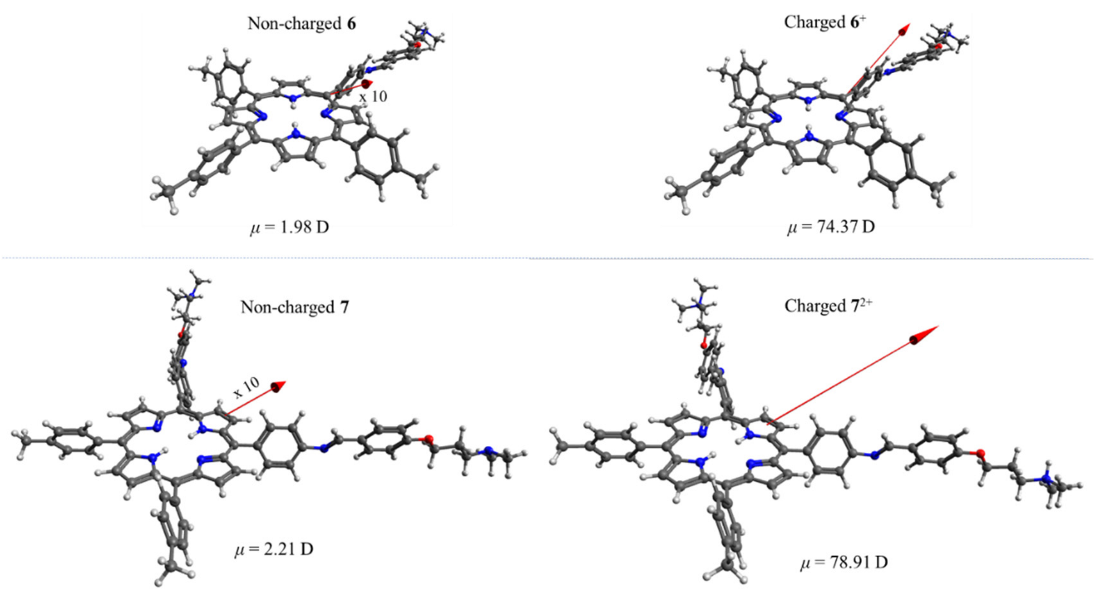

Figure S1. Optimized structure of non-charged **6** and **7** and protonated analogs **6**$^{+}$ and **7**$^{2+}$ calculated for ground state by using DFT at the B3LYP/6-31G(d) level and red arrows indicate the calculated relative magnitude and orientation of permanent dipole moment (μ).

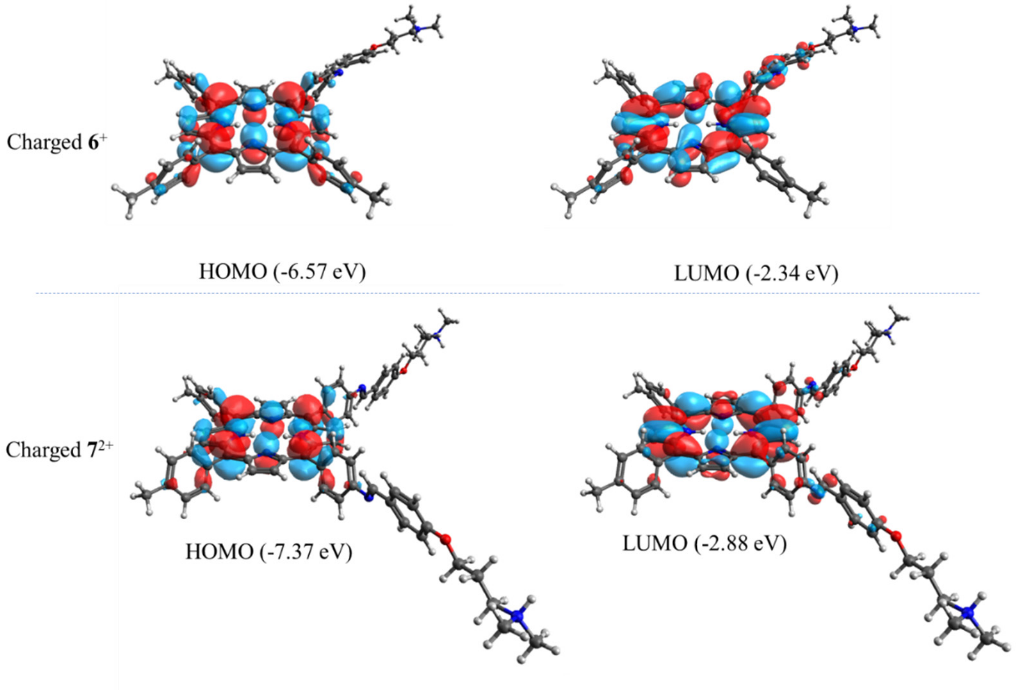

Figure S2. Highest occupied molecular orbital (HOMO) and lowest unoccupied molecular orbital (LUMO) of charged porphyrins **6**$^{+}$ (up) and **7**$^{2+}$ (down). All calculations were performed by DFT at the CAM-B3LYP/6–31 + G(d) level using Gaussian 09.

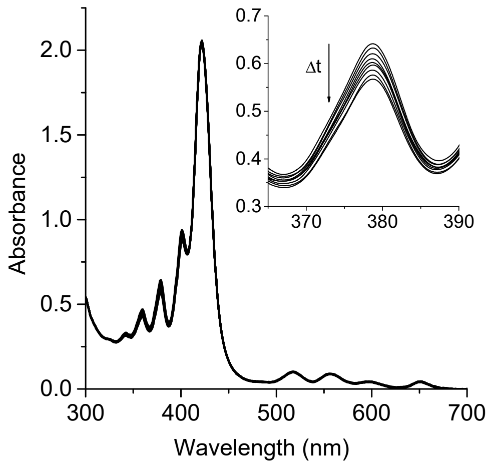

Figure S3. Absorption spectral changes during the photooxidation of DMA sensitized by **6** in DMF at different irradiation times (Δt = 120 s), λ_{irr} = 517 nm.

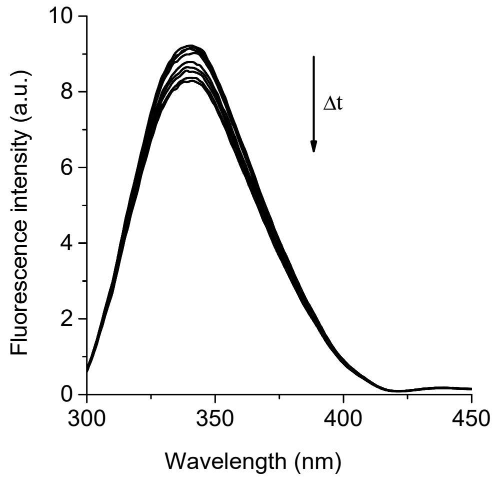

Figure S4. Fluorescence spectral (λ_{exc} = 290 nm) changes during the photodecomposition of Trp sensitized by **6** in DMF at different irradiation times (Δt = 300 s), λ_{irr} = 517 nm.

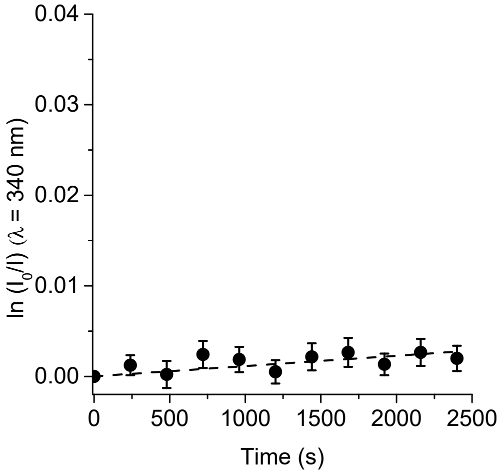

Figure S5. First-order plots for the stability of Trp in the presence of Lugol (20 μM) in DMF/10% water in the dark.

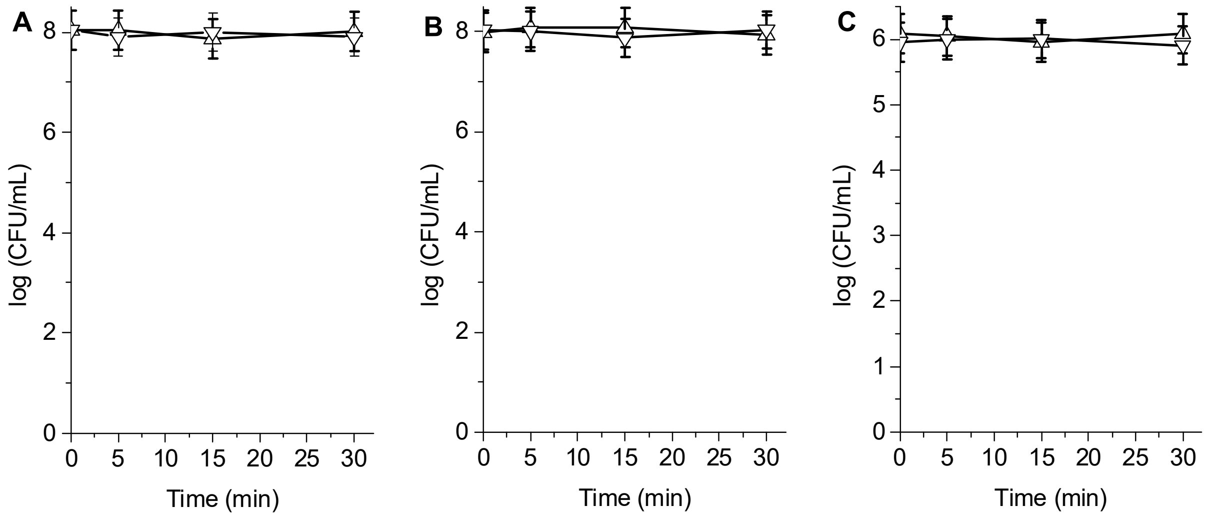

Figure S6. Survival curves of (A) *S. aureus* (~10^8 CFU/mL), (B) *E. coli* (~10^8 CFU/mL) and (C) *C. albicans* (~10^6 CFU/mL) treated with porphyrin **6** (▽) and **7** (△) for different times in the dark at 37 °C. Cultures of *S. aureus*, *E. coli* and *C. albicans* were treated with 1.0, 10.0 and 5.0 μM porphyrin, respectively.

7. References

1. Ballatore, M. B.; Milanesio, M. E.; Fujita, H.; Lindsey, J. S.; Durantini, E. N. Bacteriochlorin-

bis(spermine) conjugate affords an effective photodynamic action to eradicate microorganisms. *J. Biophotonics* **2020**, *13*, e201960061.

2. Scanone, A. C.; Gsponer, N. S.; M. G. Alvarez, M. G.; Durantini E. N. Photodynamic properties and photoinactivation of microorganisms mediated by 5,10,15,20-tetrakis(4-carboxyphenyl)porphyrin covalently linked to silica-coated magnetite nanoparticles. *J. Photochem. Photobiol. A: Chem.* **2017**, *346*, 452-461.

3. Agazzi, M. L.; Durantini, J. E.; Gsponer, N. S.; Durantini, A. M.; Bertolotti, S. G.; Durantini, E. N. Light-harvesting antenna and proton-activated photodynamic effect of a novel BODIPY-fullerene C_{60} dyad as potential antimicrobial agent. *ChemPhysChem* **2019**, *20*, 1110-1125.

4. Yanai, T.; Tew, D. P.; Handy, N. C. A new hybrid exchange–correlation functional using the Coulomb-attenuating method (CAM-B3LYP). *Chem. Phys. Lett.* **2004**, *393*, 51-57.

5. Hanwell, M. D.; Curtis, D. E.; Lonie, D. C.; Vandermeersch, T.; Zurek, E.; Hutchison, G. R. Avogadro: an advanced semantic chemical editor, visualization, and analysis platform. *J. Cheminformatics* **2012**, *4*, 1-17.

6. Reynoso, E.; Quiroga, E. D.; Agazzi, M. L.; Ballatore, M. B.; Bertolotti, S. G.; Durantini, E. N. Photodynamic inactivation of microorganisms sensitized by cationic BODIPY derivatives potentiated by potassium iodide. *Photochem. Photobiol. Sci.* **2017**, *16*, 1524-1536.

7. Durantini, E. N.; Silber, J. J. Synthesis of 5-(4-acetamidophenyl)-10,15,20-tris(4-substituted phenyl) porphyrins using dipyrromethanes. *Synth. Commun.* **1999**, *29*, 3353-3368.

8. Fungo, F.; Otero, L. A.; Sereno, L.; Silber, J. J.; Durantini, E. N. Synthesis of porphyrin dyads with potential use in solar energy conversion. *J. Mater. Chem.* **2000**, *10*, 645-650.

9. Lazzeri, D.; Durantini, E. N. Synthesis of *meso*-substituted cationic porphyrins as potential photodynamic agents. *ARKIVOC* **2003**, *10*, 227-239.

10. Mosinger, J.; Janošková, M.; Lang, K.; Kubat, P. Lightinduced aggregation of cationic porphyrins. *J. Photochem. Photobiol. A: Chem.* **2006**, *181*, 283-289.
